# Weed-infecting viruses in a tropical agroecosystem present different threats to crops and evolutionary histories

**DOI:** 10.1371/journal.pone.0250066

**Published:** 2021-04-28

**Authors:** Minor R. Maliano, Mônica A. Macedo, Maria R. Rojas, Robert L. Gilbertson

**Affiliations:** 1 Department of Plant Pathology, University of California, Davis, California, United States of America; 2 Federal Institute of Education, Science and Technology Goiano, Campus Urutaí, Goias, Brazil; Washington State University, UNITED STATES

## Abstract

In the Caribbean Basin, malvaceous weeds commonly show striking golden/yellow mosaic symptoms. Leaf samples from *Malachra* sp. and *Abutilon* sp. plants with these symptoms were collected in Hispaniola from 2014 to 2020. PCR tests with degenerate primers revealed that all samples were infected with a bipartite begomovirus, and sequence analyses showed that *Malachra* sp. plants were infected with tobacco leaf curl Cuba virus (TbLCuCV), whereas the *Abutilon* sp. plants were infected with a new bipartite begomovirus, tentatively named Abutilon golden yellow mosaic virus (AbGYMV). Phylogenetic analyses showed that TbLCuCV and AbGYMV are distinct but closely related species, which are most closely related to bipartite begomoviruses infecting weeds in the Caribbean Basin. Infectious cloned DNA-A and DNA-B components were used to fulfilled Koch’s postulates for these diseases of *Malachra* sp. and *Abutilon* sp. In host range studies, TbLCuCV also induced severe symptoms in *Nicotiana benthamiana*, tobacco and common bean plants; whereas AbGYMV induced few or no symptoms in plants of these species. Pseudorecombinants generated with the infectious clones of these viruses were highly infectious and induced severe symptoms in *N*. *benthamiana* and *Malachra* sp., and both viruses coinfected *Malachra* sp., and possibly facilitating virus evolution via recombination and pseudorecombination. Together, our results suggest that TbLCuCV primarily infects *Malachra* sp. in the Caribbean Basin, and occasionally spills over to infect and cause disease in crops; whereas AbGYMV is well-adapted to an *Abutilon* sp. in the Dominican Republic and has not been reported infecting crops.

## Introduction

The genus *Begomovirus* (family *Geminiviridae*) is comprised of a large and diverse group of plant viruses that possess a circular, single-stranded (ss) DNA genome encapsidated into twin quasi-icosahedral virions (18 x 30 nm) [1–3]. These viruses infect dicotyledonous plants and cause numerous economically important diseases of fiber, fruit, ornamental and vegetable crops, mostly in tropical and subtropical regions of the world [4]. Begomoviruses are transmitted, plant-to-plant, by whiteflies of the *Bemisia tabaci* cryptic species complex [3,5–7].

The genome of begomoviruses is composed of either a single genomic DNA of ~ 2.8 kb (monopartite) or two ~2.6 kb DNA components (bipartite), designated as DNA-A and DNA-B [1–3]. The genomic DNA of monopartite begomoviruses is homologous to the DNA-A component of bipartite begomoviruses, and both are organized with overlapping virion (v)-sense and complementary (c)-sense genes transcribed in a bidirectional manner from an intergenic region (IR), which contains the *cis*-acting elements involved in replication and gene expression (e.g., replication-associated protein [Rep] high affinity binding sites [iterons], the origin of replication [*ori*] and two bidirectional RNA polymerase II promoters) [2]. In bipartite begomoviruses, an ~200 nucleotide (nt) noncoding sequence is shared between cognate DNA-A and DNA-B components, and this common region (CR) maintains the specificity of replication for these components. Otherwise, the sequences of the DNA-A and DNA-B components are different, and both components are needed for induction of typical disease symptoms [1,2,8].

In terms of begomovirus evolution, continental drift is believed to have separated ancestral monopartite and bipartite begomoviruses, resulting in the predominance of monopartite begomoviruses in the Old World (OW) and bipartite ones in the New World (NW). The subsequent independent diversification and evolution of OW and NW begomoviruses involved different combinations of mutation, recombination and acquisition and modification of foreign DNAs [1,4,9–11]. For OW monopartite begomoviruses, acquisition of satellite DNAs has played a major role in evolution, whereas acquisition and modification of the DNA-B component was essential for bipartite begomoviruses, and allowed for pseudorecombination to act as an additional mechanism of evolution [1,8,12–17]. Furthermore, the emergence of new begomoviruses has been facilitated by the global spread of the highly polyphagous *B*. *tabaci* species MEAM1, which can introduce mixtures of viral components/genomic DNAs into a diversity of plant species [4,6,14,18]. Finally, human activities have led to the long-distance intercontinental movement of numerous begomoviruses, blurring the geographic separation of OW and NW begomoviruses [6].

The remarkable diversification of begomoviruses has been reflected in the appearance of diseases of crop and non-cultivated plants in tropical and subtropical regions worldwide. In these agroecosystems, it is common to observe non-cultivated plants (mostly weeds) showing striking golden/yellow mosaic symptoms, which are commonly associated with begomovirus infection. In the Caribbean Basin and other parts of Latin America, non-cultivated plants with these symptoms have been reported from species in the families Asteraceae, Capparaceae, Convolvulaceae, Euphorbiaceae, Fabaceae, Malvaceae, Nyctaginaceae and Solanaceae [19–35]. Importantly, characterization of begomoviruses associated with these diseases has revealed substantial genetic divergence from viruses that cause economically important crop diseases, although there are some exceptions such as the golden/yellow mosaic symptoms of *Malachra alceifolia* associated with tobacco leaf curl Cuba virus (TbLCuCV) infection in Jamaica (JM) [36], and mosaic and crumpling symptoms of *Nicandra physaloides* infected with tomato severe rugose virus in Brazil [37]. This suggests that begomoviruses infecting crops and weeds have co-evolved independently with their hosts, with the practical implication that most of these symptomatic weeds are not major sources of inoculum for crop-infecting begomoviruses. However, these begomovirus-infected weeds can serve as a mixing vessels for evolution of viruses with the potential to infect crops [34,38].

The family Malvaceae, commonly referred to as mallows, is comprised of >4225 species of annual and perennial plants [39,40]. Members of this family are distributed worldwide, and occur in temperate, tropical and subtropical regions [40,41]. Some species are important crops, such as cotton and okra [41]; others are grown as ornamentals or for medicinal purposes; and others are considered invasive weeds, e.g., *Abutilon* spp., *Sida* spp. and *Malachra* spp. [41]. Moreover, these malvaceous weeds are commonly infected by begomoviruses and develop striking golden/yellow mosaic symptoms [25,33,34,36,42–47].

As part of a long-term study to characterize begomoviruses causing golden/yellow mosaic symptoms in weeds and assesses the potential of these viruses to cause diseases of crop plants in the Dominican Republic (DO), we describe here the molecular and biological properties of two bipartite begomoviruses associated with these symptoms in *Malachra* sp. and *Abutilon* sp. plants on Hispaniola. Sequence and phylogenetic analyses together with infectivity studies with infectious clones were used to establish that the symptoms in *Malachra* sp. were caused by the crop-infecting bipartite begomovirus TbLCuCV, whereas those in *Abutilon* sp. were caused by a new species of weed-infecting begomovirus for which the name Abutilon golden yellow mosaic virus (AbGYMV) is proposed. Host range experiments showed that TbLCuCV also induced moderate to severe disease symptoms in *Nicotiana benthamiana*, tobacco (*N*. *tabacum*) and common bean plants (*Phaseolus vulgaris*) plants. In contrast, AbGYMV induced mild or no symptoms in these plants, indicating a high degree of adaptation to *Abutilon* sp. from the DO and low potential to cause crop diseases. TbLCuCV and AbGYMV are closely related species in the Abutilon mosaic virus (AbMV) lineage of NW begomoviruses and we present evidence that recombination and pseudorecombination play a role in the evolution of these viruses.

## Materials and methods

### Sample collection and DNA extraction

Leaf samples were collected from malvaceous weeds with golden/yellow mosaic symptoms (S1 Fig) in disturbed areas around agricultural fields at five locations on the island of Hispaniola from 2014 to 2020 (S2 Fig). Sample M1 was collected in Port-au-Prince, Haiti (HT) in 2014; samples M2-M4 were collected from three locations in the DO in 2016: Juan Gomez (sample M2), Manzanillo (sample M3) and Cerro Gordo (sample M4); and samples M5-M10 were collected from two locations in the DO in 2020: Cerro Gordo (samples M5-M8) and Monte Cristi (samples M9-M10) (S2 Fig). Note that Monte Cristi is ~1 to 2 km from the border with HT, in the northern part of Hispaniola.

Leaf pieces (~2 cm^2^) of the M1 sample were ground in buffer and sap was applied to Agdia absorption strips (Agdia, Elkhart, IN, USA) as described by Melgarejo et al. (2014) [34]. The strips were dried overnight at room temperature and transported to the University of California at Davis (UC Davis). For samples M2 to M10, leaf tissue was squashed onto FTA Elute Micro Cards (Whatman). The FTA cards were kept at room temperature and transported to UC Davis. Total genomic DNA was extracted from the dried plant sap on the absorption strips or FTA cards as described by Dellaporta et al. (1983).

### DNA barcode identification of malvaceous weeds

To determine the identity of the malvaceous weeds from which the samples were collected, the internal transcribed spacer (ITS) of the nuclear ribosomal DNA was amplified by the polymerase chain reaction (PCR) with the primer pair ITS-p5/ITS-u4 [48]. PCR-amplified fragments (~800 bp) were purified with the QIAquick gel extraction kit (Qiagen, Germantown, MD) and directly sequenced with the ITS-p5 and ITS-u4 primers at the UC Davis DNA Sequencing Facility.

### Detection, characterization and cloning of begomovirus DNA components

To detect begomovirus DNA-A and DNA-B components, PCR tests were performed with the degenerate primer pairs PAL1v1978/PAR1c496 and PCRc1/PLB1v2040, respectively [49]. PCR-amplified fragments were purified with the QIAquick gel extraction kit and directly sequenced with the PAL1v1978/PAR1c496 and PCRc1/PLB1v2040 primers.

To estimate the number and genetic diversity of begomovirus DNAs present in the samples and to identify single-cutting restriction enzymes for obtaining full-length clones, restriction fragment length polymorphism (RFLP) analyses of circular DNAs generated by rolling circle amplification (RCA) with Φ-29 DNA polymerase (TempliPhi; GE Healthcare, Piscataway, NJ) were performed [50]. The RCA products were first digested with the four-base-cutting enzyme *Msp*I to generate RFLPs for estimating the number of begomovirus DNA components infecting the samples. Next, RCA products were digested with selected six-base-cutting enzymes to identify sites in each DNA component for obtaining full-length clones. The linearized DNA components were ligated into pGEM11Z (+) (Promega Corp., Madison, WI) or pSL1180 (Amersham Biosciences) digested with the appropriate enzyme. Recombinant plasmids having the full-length DNA-A and DNA-B components were identified by restriction enzyme digestion and DNA sequence analyses. Based upon sequencing and RCA results, the begomovirus isolates from the M1-M4 samples were selected for further studies. Thus, full-length DNA-A and DNA-B clones were obtained from sample M1 (in plasmids pM-GV1-HT-A and pM-GV1-HT-B), sample M2 (in plasmids pM-GV2-JG-A and pM-GV2-JG-B), sample M3 (in plasmids pM-GV3-M-A and pM-GV3-M-B) and sample M4 (in plasmids pAb-GV4-CG-A and pAb-GV4-CG-B).

### Sequence analyses

The complete sequences of the cloned full-length DNA-A and DNA-B components of the bipartite begomoviruses from samples M1-M4 were determined and analyzed with Vector NTI advance software (Invitrogen, Carlsbad, CA). A BLASTn search was initially performed to identify sequences in GenBank with highest identities [51]. Pairwise nt sequence alignments were performed with MUSCLE within the Species Demarcation Tool (SDT) v.1.2, and with full-length DNA-A and DNA-B sequences of the ten begomoviruses with the highest identities revealed by the BLASTn search [52,53]. The Vector NTI advance software was used to make more extensive comparisons, including individual open reading frames (ORFs) and non-translated regions (NTRs) from both components. The *cis*-acting elements involved in begomovirus replication (i.e., iterons and the Rep iteron-related domains [IRDs]) were identified as described in Argüello-Astorga and Ruiz-Medrano (2001) [68].

### Phylogenetic analyses

For the phylogenetic analyses, we used the complete nt sequences of the DNA-A and DNA-B components of: (i) the bipartite begomoviruses from the M1-M4 samples; (ii) TbLCuCV isolates from Cuba (CU) (the DNA-A and DNA-B of one isolate and the DNA-A of two other isolates); (iii) the ten most identical viruses revealed by the BLASTn search; and (iv) selected viruses representing the AbMV, Brazil, squash leaf curl virus (SLCuV), bean golden yellow mosaic virus (BGYMV) and Boerhavia golden mosaic virus (BoGMV) lineages of NW begomoviruses. Multiple sequence alignments (MSA) for the DNA-A and DNA-B component sequences were generated with the MAFFT algorithm implemented in the Guidance2 Server [54,55]. The alignment quality was analyzed, and unreliable regions (poorly aligned) were removed with the GUIDANCE algorithm [54]. The resulting alignments were then exported as Nexus files. Phylogenetic trees were constructed with a Bayesian inference and Markov chain Monte Carlo (MCMC) simulation implemented in MrBayes V3.2 [56]. The best-fit model of nt substitution for each data set was determined with the program MrModeltest V2.2 [57]. The analyses were carried out by running 2,000,000 generations and sampling at every 100 generations, resulting in 20,000 trees. The first 10% of samples were discarded as a burn-in. Trees were visualized with Archaeopteryx tree viewer and exported in Newick format [58]. Trees were manually edited with MEGA X [59]. The DNA-A and DNA-B phylogenetic trees were rooted with the sequences of the genomic DNA of the OW monopartite begomovirus tomato yellow leaf curl virus (TYLCV) and the DNA-B component of the OW bipartite begomovirus African cassava mosaic virus (ACMV), respectively.

### Recombination analysis

Preliminary datasets of complete sequences of 584 DNA-A and 240 DNA-B components were assembled. This included the complete nt sequences of the DNA-A and DNA-B components of: (i) the bipartite begomoviruses from the M1-M4 samples; (ii) the TbLCuCV isolates from CU; and (iii) sequences of selected viruses retrieved from GenBank. SDT and the Recombination Detection Program version 4.0 (RDP4) [60] were used to remove sequences that were identical or having nt sequence identities <70%. Final datasets of complete sequences of 488 DNA-A and 201 DNA-B components were used for recombination analyses. MSA were generated with MUSCLE within MEGA X [59,61], and the alignments were manually edited and exported as FASTA files. Detection of recombination breakpoints and identification of potential parental viruses were performed with RDP4. The recombination analysis was performed with default settings and a Bonferroni-corrected *p*-value cut-off of 0.05. Only recombination events detected with three or more methods coupled with significant phylogenetic support were considered *bona fide* events.

### Production of multimeric clones and agroinoculation systems

Based upon sequencing and RCA-RFLP results, the begomovirus isolates from the M1 and M4 samples were selected for infectivity studies. Here, multimeric clones of the DNA-A and DNA-B components were generated in the pCAMBIA 1300 binary vector [62]. For the DNA-A component of the M1 isolate, an ~1.6 kb *Sal*I-*Hin*dIII fragment containing the CR was cloned into pCAMBIA 1300 to generate the 0.6-mer pM-GV1-HT-A0.6. The full-length DNA-A monomer was released with *Hin*dIII from pM-GV1-HT-A and cloned into the *Hin*dIII-digested pM-GV1-HT-A0.6 to generate pM-GV1-HT-A1.6. For the DNA-B component, an ~1.5 kb *Sac*I-*Xba*I fragment containing the CR was cloned into pCAMBIA 1300 to generate the 0.6-mer pM-GV1-HT-B0.6. The full-length DNA-B monomer was released with *Xba*I from pM-GV1-HT-B and cloned into the *Xba*I-digested pM-GV1-HT-B0.6 to generate pM-GV1-HT-B1.6.

For the DNA-A component of the M4 isolate, an ~2.1 kb *Eco*RI fragment containing the CR was cloned into pCAMBIA 1300 to generate the 0.8-mer pAb-GV4-CG-A0.8. The full-length DNA-A monomer was released with *Bgl*II from pAb-GV4-CG-A and cloned into the *Bgl*II-digested pAb-GV4-CG-A0.8 to generate pAb-GV4-CG-A1.8. For the DNA-B component, an ~1.2 kb *Bam*HI fragment containing the CR was cloned into pCAMBIA 1300 to generate the 0.5-mer pAb-GV4-CG-B0.5. The full-length DNA-B monomer was released with *Bam*HI from pAb-GV4-CG-B and cloned into the *Bam*HI-digested pAb-GV4-CG-B0.5 to generate pAb-GV4-CG-B1.5. Recombinant plasmids having the multimeric clones were identified for each component by restriction enzyme digestion. Selected plasmids with the confirmed multimeric clones were then transformed into electrocompetent *Agrobacterium tumefaciens* cells (strain C58C1) by electroporation.

### Infectivity and host range experiments

The infectivity of the cloned DNA components was first assessed by agroinoculation of *N*. *benthamiana* plants at the three- to five-leaf-stage (3 weeks old). Plants were agroinoculated with mixtures of *A*. *tumefaciens* cell suspensions (optical density of 600 nm = 1.0) of strains containing binary plasmids with the multimeric DNA-A and DNA-B clones of the M1 and M4 isolates by needle puncture inoculation of the stem just beneath the shoot apex [63]. A host range experiment was conducted by agroinoculation of *Malachra* sp. and *Abutilon indicum* and *Abutilon* sp. plants (from seeds collected from the DO), *N*. *benthamiana*, *N*. *tabacum* cvs. Havana and Turkish, *N*. *glutinosa*, *Solanum lycopersicum* cv. Glamour, *Datura stramonium*, *Chenopodium amaranticolor*, *Cucurbita maxima* cv. Sugarpie and *P*. *vulgaris* cv. Topcrop plants at the two- to five-leaf-stage (3 weeks old). The negative control was equivalent plants of these species agroinoculated with cell suspensions of an *A*. *tumefaciens* strain containing the empty vector (pCAMBIA 1300). Infectivity in pepper (*Capsicum annuum* cv. Cayenne) was determined by particle bombardment inoculation with the multimeric DNA-A and DNA-B clones of each isolate, as described in Paplomatas et al. (1994). The positive control was equivalent plants bombarded with the multimeric DNA-A and DNA-B clones of the pepper-infecting bipartite begomovirus pepper huasteco yellow vein virus (PHYVV), whereas the negative control was plants bombarded with gold particles alone.

Inoculated plants were maintained in a controlled environment chamber as described by Melgarejo et al. (2014) [34]. Symptom development was assessed visually and recorded at 14 days post inoculation (dpi). In selected symptomatic and all non-symptomatic plants, the presence of viral DNA in newly emerged (non-inoculated) leaves was determined by PCR tests with component-specific primers for each virus (S1 Table).

### Pseudorecombination experiments with cloned DNA components of TbLCuCV and AbGYMV

These experiments were performed by agroinoculating *N*. *benthamiana* and *Malachra* sp. plants with all combinations of the multimeric DNA-A or DNA-B clones of the M1 and M4 isolates. The negative control was equivalent plants agroinoculated with the empty vector. Inoculated plants were maintained in a controlled environment chamber, and symptom development was assessed as previously described. The presence of the inoculated DNA-A and DNA-B components in newly emerged leaves was determined by PCR tests with component-specific primers for each virus (S1 Table).

### Co-infection studies with TbLCuCV and AbGYMV

For these experiments, *Malachra* sp. seedlings were co-agroinoculated with a mixture of cell suspensions of *A*. *tumefaciens* strains containing binary plasmids with the multimeric DNA-A and DNA-B clones of the M1 and M4 isolates. Control plants were agroinoculated with the DNA-A and DNA-B components of the M1 or M4 isolates or with the empty vector. Inoculated plants were maintained in a controlled environment chamber, and symptom development was assessed as previously described. The presence of the inoculated DNA components in newly emerged leaves was determined by PCR tests with component-specific primers for each virus (S1 Table).

## Results

### DNA barcode identification of malvaceous weeds

Samples of leaves with golden/yellow mosaic symptoms typical of begomovirus infection were collected from two types of malvaceous weeds: a large plant (height of 1 to 2 m) with malva-like leaves (samples M1 from HT and M2 and M3 from the DO, S1A Fig), and a smaller shrub-like plant (height of 0.5 to 1 m) with heart-shaped leaves (samples M4 to M10 from the DO, S1B Fig). In PCR tests with the plant-specific ITS primer pairs, the expected-size ~0.8 kb fragment was amplified from samples M1-M10. BLASTn analyses revealed that the ITS sequences from samples M1-M3 (large-size plant) had highest identities (94%) with those of *Malachra* spp., whereas those of samples M4-M10 (smaller-size plant) had highest identities (94%) with those of *Abutilon* spp. Thus, these results indicated that the large malva-like weed was *Malachra* sp., whereas the smaller shrub-like malvaceous weed was *Abutilon* sp.

### Detection, characterization and cloning of begomovirus DNA components

In PCR tests with degenerate DNA-A and DNA-B primer pairs, the expected-sized ~1.1- and ~0.5-kb DNA fragments, respectively, were amplified from samples M1-M10, indicating infection with a bipartite begomovirus. Based upon BLASTn analyses, the sequences of the PCR-amplified DNA-A and DNA-B fragments from the M1-M3 samples were >95% identical to each other and had highest identities (>96% for DNA-A and >94% for DNA-B sequences) with sequences of isolates of TbLCuCV from CU and JM. The sequences of the PCR-amplified DNA-A and DNA-B fragments from the M4-M10 samples were >95% identical to each other and had highest identities (~86% for DNA-A and ~85% for DNA-B sequences) with sequences of the TbLCuCV isolates and various weed-infecting begomoviruses from the Caribbean Basin. Furthermore, *Msp*I digestion of the RCA products generated from these samples yielded fragments that totaled ~5.2 kb, consistent with infection with a single bipartite begomovirus. Taken together, these results suggest that the *Malachra* sp. plants with golden/yellow mosaic symptoms were associated with variants of TbLCuCV, whereas the *Abutilon* sp. plants with these symptoms were associated with variants of a putative new begomovirus species.

The M1-M3 isolates from *Malachra* sp. and the M4 isolate from *Abutilon* sp. were selected for further characterization. For the M1 sample, full-length (~2.7 kb) linear ds DNA-A and DNA-B components were generated with RCA products with *Hin*dIII and *Xba*I, respectively, and cloned; for samples M2 (Juan Gomez) and M3 (Manzanillo) with *Hin*dIII and *Sal*I, respectively; and for sample M4 (Cerro Gordo) with *Bgl*II for both components.

### Genomic properties of full-length begomovirus DNA components

The complete sequences of the cloned full-length DNA-A and DNA-B components of the M1-M4 isolates were determined. The DNA-A and DNA-B components of the M1 isolate from HT are 2,611 (GenBank accession number MH514009) and 2,567 nt (GenBank accession number MH514010), respectively; those of the M2 and M3 isolates from Juan Gomez (DO) and Manzanillo (DO) are 2,610 (GenBank accession number MK059404) and 2,565 nt (GenBank accession number MK059405) and 2,609 (GenBank accession number MK059402) and 2,565 nt (GenBank accession number MK059403), respectively; and those of the M4 isolate from Cerro Gordo (DO) are 2,638 (GenBank accession number MH514011) and 2,585 nt (GenBank accession number MH514012), respectively.

The genome organization of the DNA-A and DNA-B components of the begomovirus isolates of the M1-M4 samples is typical of NW bipartite begomoviruses, i.e., the DNA-A is ~2.6 kb and encodes five ORFs, one in the v-sense strand (AV1) encoding the capsid protein (CP), and four in the c-sense strand (AC1, AC2, AC3, and AC4) encoding the Rep, the transcriptional activator protein (TrAP), the replication enhancer (REn) and the AC4 protein, respectively. Additionally, the CP and REn amino acid (aa) sequences of all four isolates possess the N- and C-terminal motifs PWRpMAGT and AVRFATdR (lowercase indicates variable aa residues), respectively, which are characteristic of NW begomoviruses [34,64,65]. The DNA-B components has two ORFs, one in the v-sense strand (BV1) that encodes the nuclear shuttle protein (NSP), and one on the c-sense strand (BC1) encoding the movement protein (MP).

SDT analysis of the sequences of the complete DNA-A component of the M1-M3 isolates from *Malachra* sp. revealed highest identities (>96%) with isolates of TbLCuCV from CU (S2 Table). Similar results were obtained for the complete DNA-B component sequences, i.e., identities of >94% with an isolate of TbLCuCV from CU. Comparisons made with the nt and aa sequences of individual ORFs revealed similar identities (>94%) across all ORFs, whereas CR identities range from ≥92 to 98% and identities for the hypervariable region (HVR) of the DNA-B component range from ≥91 to 93% (S2 Table). The next highest identities for the DNA-A component sequence were with NW bipartite begomoviruses from Latin America, including tobacco mottle leaf curl virus (87%), Sida yellow mottle virus (SiYMoV) (87%) and Wissadula golden mosaic virus (WGMV) (87%) (S2 Table). In the case of the DNA-B component sequence, the next highest identities were with the M4 isolate from *Abutilon* sp. (82%) and WGMV (81%) (S2 Table). These results confirmed that *Malachra* sp. plants with golden/yellow mosaic symptoms from which the M1-M3 samples were collected were infected with variants of TbLCuCV. These new isolates are named tobacco leaf curl Cuba virus-[Haiti:2014] (TbLCuCV-[HT:14]), tobacco leaf curl Cuba virus-[Dominican Republic:Juan Gomez:2016] (TbLCuCV-[DO:JG:16]) and tobacco leaf curl Cuba virus-[Dominican Republic:Manzanillo:2016] (TbLCuCV-[DO:M:16]).

The SDT analysis performed with the complete DNA-A component sequence of the M4 isolate from *Abutilon* sp. revealed highest identities (85 to 86%) with those of the isolates of TbLCuCV from Hispaniola (present study) and CU and slightly lower identities with NW weed-infecting bipartite begomoviruses from Latin America, including Jatropha mosaic virus (85%), WGMV (85%) and Sida golden mosaic virus (84%) (S3 Table). Given that the current species demarcation value for new begomovirus species is <91% nt sequence identity for the complete DNA-A sequence (Brown et al. 2015), the M4 isolate represents a new bipartite begomovirus species, for which the name Abutilon golden yellow mosaic virus-[Dominican Republic:Cerro Gordo:2016] (AbGYMV-[DO:CG:16]) is proposed. For the DNA-B component sequence, the highest identities also were with those of the TbLCuCV isolates from CU (82%) and with weed-infecting viruses from the Caribbean Basin, including WGMV (81%) and SiYMoV (81%). The CR sequences had the highest identities (85–88%) with those of TbLCuCV isolates from Hispaniola and CU and with JMV (86%). The HVR of the DNA-B component was the most divergent region of the genome, having highest identities of 54 to 67% with tomato yellow leaf distortion virus and Corchorus yellow spot virus (S3 Table). Comparisons of the nt and aa sequences of individual ORFs of both components revealed a wide range of identities, some of which were lower than those expected for closely related species. For example, whereas the AC1, AC2, AC3 and BV1 ORFs had similar levels of identity for the nt and aa sequences, nt identities for the AV1 and BC1 ORFs were considerably lower (84 to 90%) than those for aa sequences (88 to 97%). In contrast, nt identities for the AC4 ORF were higher (78 to 86%) than those for aa sequences (54 to 68%) (S3 Table).

### Analyses of the CR sequences of TbLCuCV and AbGYMV

**Fig 1:**
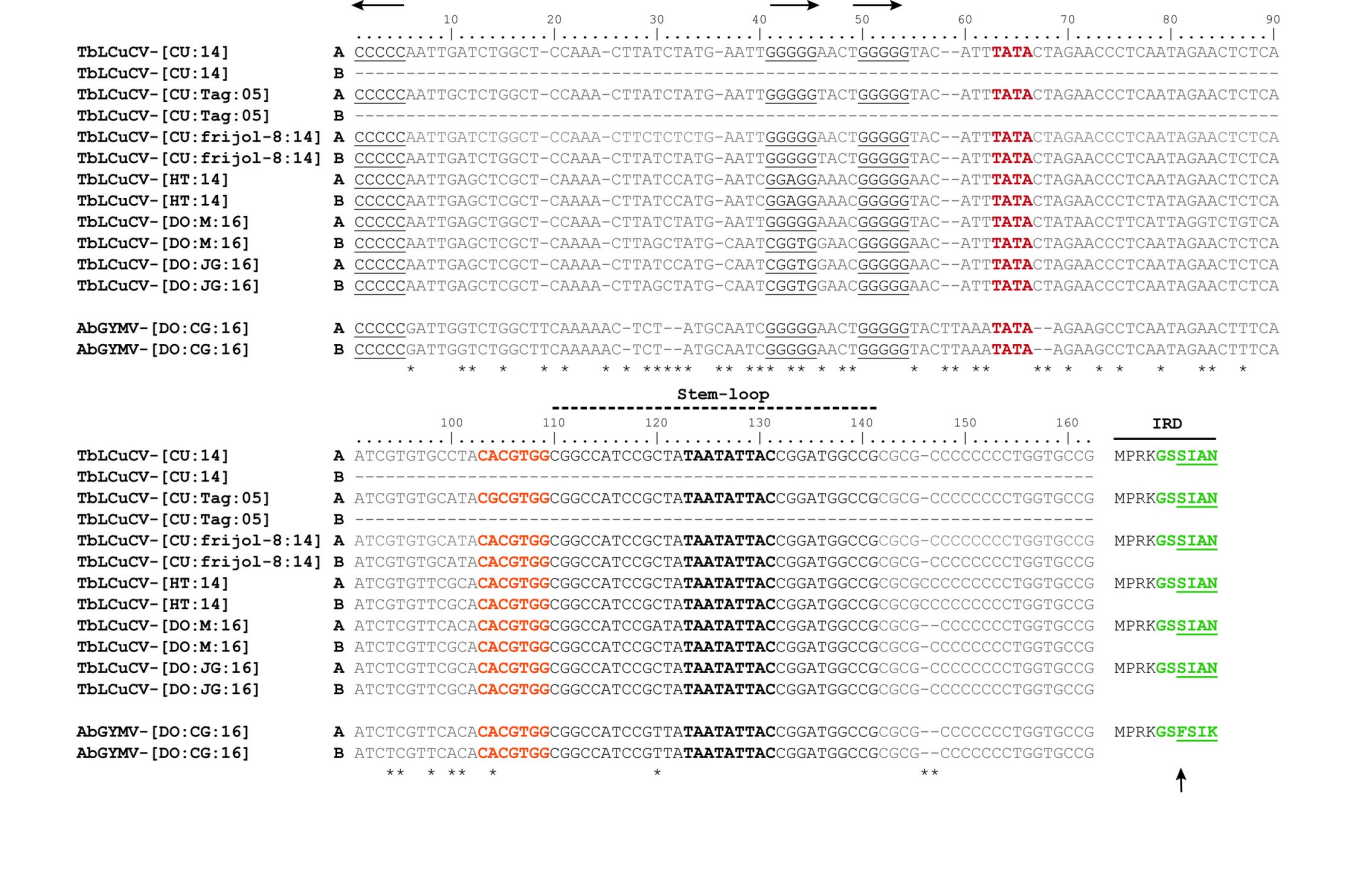
Alignment of a portion of the common region (CR) sequences of isolates of tobacco leaf curl Cuba virus (TbLCuCV) from Cuba, Haiti and the Dominican Republic (DO) and the putative new bipartite begomovirus Abutilon golden yellow mosaic virus (AbGYMV) from the DO. The replication-associated protein (Rep) high affinity binding site (two direct repeats and one inverted repeat of the core iteron) are shown in black letters and underlined, and their orientation is shown with arrows at the top of the alignment. The motif with the TATA box of the AC1 (Rep) gene is indicated in red bold red letters, whereas the G-box is represented in orange bold letters. The characteristic geminivirus stem-loop forming sequence is highlighted in black under the broken line, with the conserved geminivirus nonanucleotide sequence shown in black bold letters. The Rep iteron-related domain (IRD) located in the N-terminus of Rep, is shown to the right side of the alignment, with the putative six amino acids involved in iteron recognition indicated in green bold letters and variable positions are underlined. The highly conserved F residue in the IRD is indicated with an arrow. The numbers on top of the alignment indicate nt positions in respect to the inverted repeat, and nt differences are indicated with an asterisk. Abbreviations and GenBank accession numbers are as follows: TbLCuCV-[CU:14]: KU562963; TbLCuCV-[CU:Tag:05]: AM050143; TbLCuCV-[CU:frijol-8:14]: KX011471 and KX011472; TbLCuCV-[HT:14]: MH514009 and MH514010; TbLCuCV-[DO:M:16]: MK059402 and MK059403; TbLCuCV-[DO:JG:16]: MK059404 and MK059405 and AbGYMV-[DO:CG:16]: MH514011 and MH514012.

The DNA-A and DNA-B components of the three new isolates of TbLCuCV and the isolate of AbGYMV share a CR of ~200 nt, with identities ranging from 90–98% as expected for cognate components. These CR sequences contain the *cis*-acting elements implicated in virus replication and gene expression, e.g., the conserved stem-loop structure with the nonanucleotide sequence TAATATT↓AC and the canonical AC1 TATA box and G-box, which interact with the TATA-binding protein and the G-box transcription factor, respectively (Fig 1) [2,66,67]. The Rep high affinity-binding site (iterons) in the CR is typically organized with a direct repeat and a single upstream inverted repeat of a 5 nt core sequence motif GGN1N2N3 [68], which are specifically recognized by the iteron-related domain (IRD) located in the N-terminus region of the Rep protein, which has a canonical X_−n_…X_−2_ X_−1_ F X_1_ X_2_ X_3_ motif where X_−n_ is the first residue of the motif and F is a highly conserved phenylalanine residue [68].

The CR sequences of the TbLCuCV isolates from CU and Hispaniola have nearly identical Rep binding sites, which are organized with two direct repeats of the GGGGG core and an upstream inverted repeat CCCCC (Fig 1). The N1 position of the first iteron (GGN1GG) was variable (N1 = G, A or T), whereas the second iteron (GGGGG) was invariant among TbLCuCV isolates. The IRD of these TbLCuCV isolates is MPRK**GS****S****IAN** (key amino acids shown in bold), which is atypical in (i) lacking the highly conserved F residue, instead having a serine (S) residue (underlined) (Fig 1), and (ii) not predicted to recognize the GGGGG core sequence [68].

The Rep high affinity binding site in the CR of AbGYMV also consist of two direct repeats of the GGGGG core and an upstream inverted repeat CCCCC (Fig 1). The Rep IRD aa sequence is MPRK**GS****F****SIK**, which possesses the conserved F residue and is predicted to recognize the GGGGG core sequence [68]. Finally, it is worth noting that the portion of the TbLCuCV and AbGYMV CR sequences that lies between the inverted repeat and G-box (104 nt) was highly divergent (41%).

### Phylogenetic analyses

**Fig 2:**
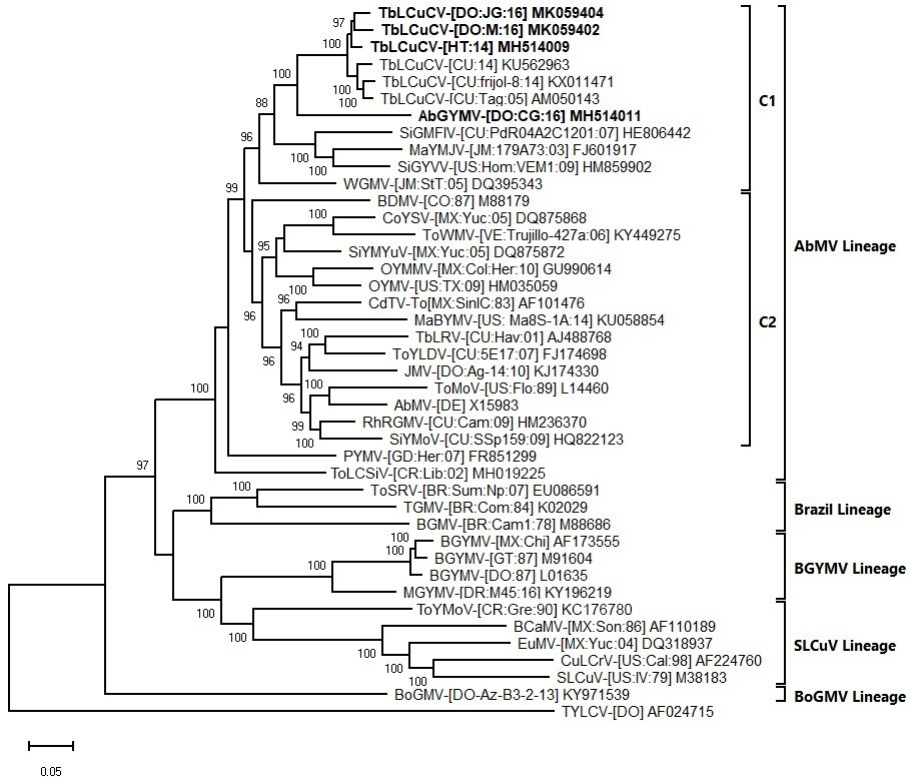
Bayesian phylogenetic consensus tree constructed with complete nucleotide sequences of the DNA-A components and showing the relationship of three isolates of tobacco leaf curl Cuba virus (TbLCuCV) from Hispaniola and the putative new bipartite begomovirus species Abutilon golden yellow mosaic virus (AbGYMV) from the Dominican Republic (shown in bold) with: (i) the most closely related begomoviruses identified based on a BLASTn search and (ii) selected begomoviruses representing the Abutilon mosaic virus (AbMV), Brazil, squash leaf curl virus (SLCuV), bean golden yellow mosaic virus (BGYMV) and Boerhavia golden mosaic virus (BoGMV) lineages of New World (NW) bipartite begomoviruses (lineages are indicated with brackets). The two major clades of the AbMV lineage, C1 and C2, are shown with inner brackets. Sequences were obtained from GenBank, and virus abbreviations are as described in Brown et al. 2015. Branch strengths were evaluated by Bayesian posterior probabilities. The phylogenetic consensus tree was rooted with the complete sequence of the genomic DNA of the Old World monopartite begomovirus tomato yellow leaf curl virus (TYLCV). The length of horizontal branches indicates the rate of substitution per nucleotide.

In the phylogenetic tree generated with the complete DNA-A sequences, the TbLCuCV isolates from Hispaniola formed a strongly supported clade with the isolates from CU. Within this clade there was evidence of genetic divergence between isolates from CU and Hispaniola, consistent with geographical separation (note that complete DNA-A sequences for TbLCuCV isolates from JM are not available) (Fig 2). In this tree, AbGYMV was placed on a distinct branch (sister clades), which was included in a larger strongly supported clade with the TbLCuCV isolates. This clade was part of the larger C1 clade of the AbMV lineage, which includes mostly weed-infecting begomoviruses from the Caribbean Basin (Fig 2), whereas the other large clade (C2) included crop- and weed-infecting begomoviruses from many countries of Latin America (Fig 2).

The phylogenetic tree generated with the complete DNA-B sequences revealed a similar overall topology, but with some notable differences. The TbLCuCV isolates from Hispaniola and CU were placed in a strongly supported clade in the AbMV lineage (S3 Fig). In contrast to the DNA-A tree, AbGYMV did not form a sister clade with the TbLCuCV isolates, but was placed together with the TbLCuCV isolates and other weed-infecting bipartite begomoviruses from the Caribbean Basin in the strongly supported C1 clade of the AbMV lineage (S3 Fig).

In the DNA-B tree, the C2 clade included viruses from North and Central America and the Caribbean Basin, whereas more distantly related viruses from South America were placed in a paraphyletic group (C3 clade) (S3 Fig). Finally, whereas the DNA-A tree clearly separates the BGYMV, Brazil, SLCuV and BoGMV lineages, these clades clustered together in a larger clade in the DNA-B tree (compare Figs 2 and S3). Taken together with the SDT analysis and sequence comparisons, the results of the phylogenetic analyses are consistent with TbLCuCV and AbGYMV representing distinct but closely related species, which are most closely related to NW bipartite begomovirus species infecting weeds in the Caribbean Basin.

### Recombination analysis

RDP4 analysis did not reveal evidence of recombination in the DNA-A and DNA-B components of the TbLCuCV isolates from Hispaniola and CU. For AbGYMV, a single recombination event of 625 nt was detected in the DNA-A component, whereas no recombination was not detected in the DNA-B component. The recombination event in the AbGYMV DNA-A component was strongly supported by six methods implemented in RDP4 (*p*-values of 9.178×10^−15^, 3.563×10^−17^, 1.059×10^−09^, 3.883×10^−10^, 1.462×10^−19^ and 9.246×10^−05^ for the RDP, GENECONV, MaxChi, Chimaera, SiScan and 3Seq recombination methods, respectively). This region spans nts 1994 to 2618, which includes the 5’ end of the AC1 ORF, the entire AC4 ORF and the left side of the CR (S4 Fig). Thus, this event is in the well-known recombination hot-spot in the begomovirus genomic DNA/DNA-A component [13,69,70], and explains the higher levels of genetic divergence detected in this region (S3 Table). The RDP4 analysis further indicated that the recombinant region was derived from an uncharacterized minor parent, whereas the major parent was tomato yellow leaf distortion virus (GenBank accession number FJ174698).

### Infectivity and host range experiments

**Fig 3:**
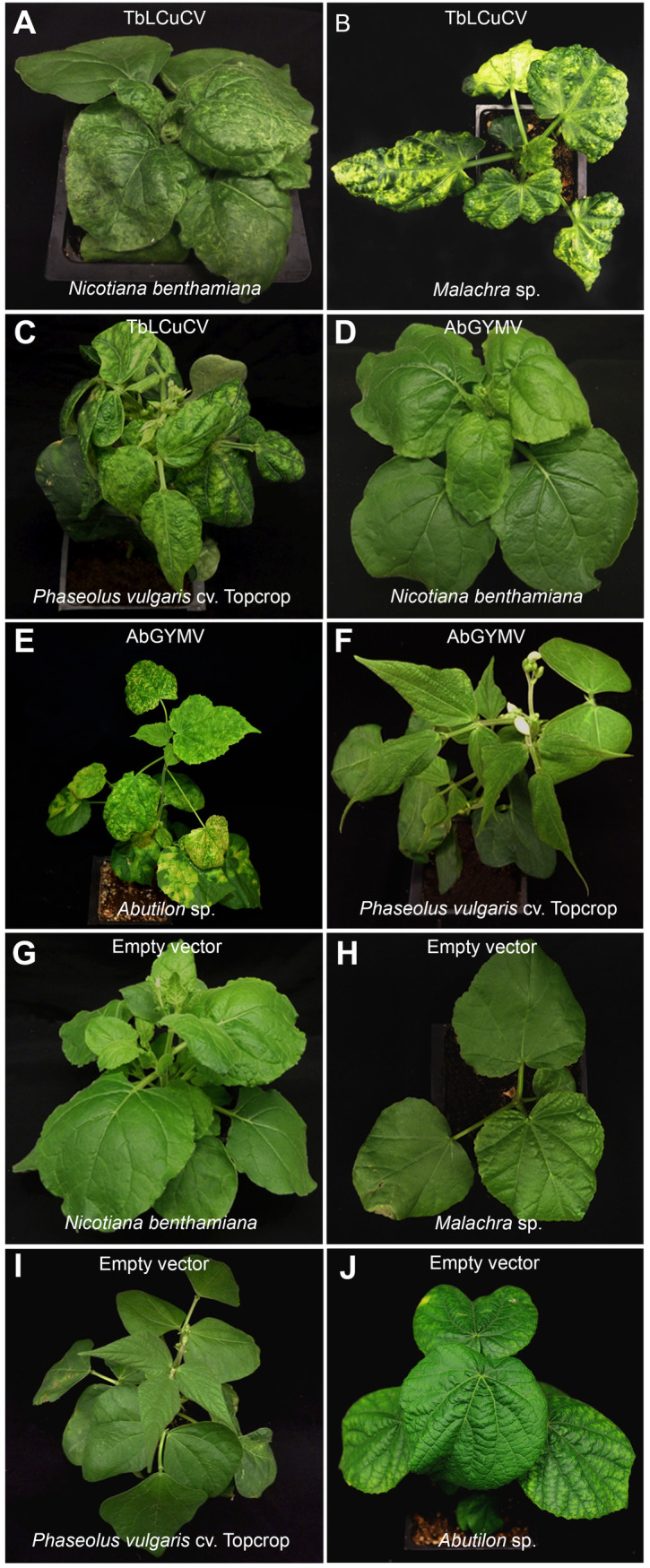
Disease symptoms induced by the infectious cloned DNA-A and DNA-B components of an isolate of tobacco leaf curl Cuba virus from Haiti (TbLCuCV-[HT:14]) in: (A) *Nicotiana benthamiana*, (B) *Malachra* sp. and (C) common bean (*Phaseolus vulgaris* cv. Topcrop); and those induced by the infectious cloned DNA-A and DNA-B components of the new bipartite begomovirus species Abutilon golden yellow mosaic virus from the Dominican Republic (AbGYMV-[DO:CG:16]) in (D) *N*. *benthamiana*, (E) *Abutilon* sp. from the DO and (F) common bean. Negative control plants agroinoculated with the empty vector: (G) *N*. *benthamiana*, (H) *Malachra* sp., (I) common bean and (J) *Abutilon* sp. Plants were photographed 14 days after agroinoculation.

In a preliminary experiment, *N*. *benthamiana* plants agroinoculated with the DNA-A and DNA-B multimeric clones of TbLCuCV-[HT:14] were stunted and newly emerged leaves showed epinasty, crumpling, deformation, mosaic and vein yellowing by 14 dpi (Table 1, Fig 3A). In the host range experiment, the infectious cloned DNA-A and DNA-B components of TbLCuCV induced stunting and golden/yellow mosaic in newly emerged leaves of all agroinoculated *Malachra* sp. plants by 14 dpi (Fig 3B). These symptoms were similar to those observed in *Malachra* sp. plants in the field in Hispaniola (S1A Fig), thereby fulfilling Koch’s postulates for the golden/yellow mosaic disease of *Malachra* sp. TbLCuCV also induced stunting and epinasty and crumpling of newly emerged leaves of agroinoculated *N*. *tabacum* (cvs. Havana and Turkish) and *N*. *glutinosa* plants, and stunting and epinasty, deformation, chlorosis and mosaic of newly emerged leaves of agroinoculated common bean (cv. Topcrop) plants by 14 dpi (Fig 3C). *D*. *stramonium* plants agroinoculated with TbLCuCV developed chlorotic spots in newly emerged leaves, whereas symptomless DNA-A and DNA-B infections were detected in some (<50%) agroinoculated tomato plants by 14 dpi (Table 1). TbLCuCV did not infect Cayenne long pepper (inoculated by particle bombardment), pumpkin and *C*. *amaranticolor* plants.

**Table 1:** Infectivity, host range and symptomatology of the infectious cloned DNA-A and DNA-B components of an isolate of tobacco leaf curl Cuba virus from Haiti (TbLCuCV-[HT:14]) and Abutilon golden yellow mosaic virus from the Dominican Republic (AbGYMV-[DO:CG:16]).

Host plant/Begomovirus species	Infectivity^a^		Symptoms^b^
	Agroinoculation	Bombardment	
*Nicotiana benthamiana*			
TbLCuCV	10/10 (100)	NT	Cr, E, Ld, M, S, Vy
AbGYMV	9/9 (100)	NT	Cr, E, S
*Nicotiana tabacum* cv. Havana			
TbLCuCV	9/9 (100)	NT	Cr, E, S
AbGYMV	9/9 (100)	NT	NS-i
*Nicotiana tabacum* cv. Turkish			
TbLCuCV	9/9 (100)	NT	Cr, E, S
AbGYMV	9/9 (100)	NT	NS-i
*Nicotiana glutinosa*			
TbLCuCV	9/9 (100)	NT	Cr, E, M, S
AbGYMV	9/9 (100)	NT	U
*Solanum lycopersicum* cv. Glamour			
TbLCuCV	4/9 (44)	NT	NS-i
AbGYMV	1/9 (10)	NT	NS-i
*Capsicum annuum* cv. Cayenne			
TbLCuCV	NT	0/9 (0)	NS-ni
AbGYMV	NT	0/9 (0)	NS-ni
*Datura stramonium*			
TbLCuCV	4/9 (44)	NT	Cs
AbGYMV	6/9 (66)	NT	NS-i
*Chenopodium amaranticolor*			
TbLCuCV	0/9 (0)	NT	NS-ni
AbGYMV	0/9 (0)	NT	NS-ni
*Cucurbita maxima* cv. Sugarpie			
TbLCuCV	0/9 (0)	NT	NS-ni
AbGYMV	0/9 (0)	NT	NS-ni
*Phaseolus vulgaris* cv. Topcrop			
TbLCuCV	10/10 (100)	NT	Ch, E, Ld, M, S
AbGYMV	17/17 (100)	NT	E
*Malachra* sp.			
TbLCuCV	12/12 (100)	NT	Cr, E, Ld, M, S
AbGYMV	1/12 (8)	NT	NS-i
*Abutilon indicum*			
TbLCuCV	0/9 (0)	NT	NS-ni
AbGYMV	0/9 (0)	NT	NS-ni
*Abutilon* sp.^c^			
AbGYMV	5/5 (100)	NT	Cr, E, M, S

^a^ Infectivity (number of infected plants/number inoculated) was determined at 14 days after inoculation based on symptom development and detection of viral DNA components in newly emerged leaves by PCR tests with component-specific primer pairs. Percentage of plants infected is indicated in parentheses; NT = not tested. Data represents a total of three independent experiments, except for *Abutilon* sp., for which a single experiment was performed.

^b^ Abbreviations: Ch, chlorosis; Cr, crumpling; Cs, chlorotic spots; E, epinasty; Ld, leaf deformation; M, mosaic/mottle; NS-i, no symptoms-infected; NS-ni, no symptoms-not infected; S, stunting; U, upward leaf curling and Vy, vein yellowing.

^c^ *Abutilon* sp. plants were derived from seeds collected from plants in the Dominican Republic.

*N*. *benthamiana* plants agroinoculated with the multimeric cloned DNA-A and DNA-B components of AbGYMV were stunted and developed mild symptoms of epinasty and crumpling in newly emerged leaves and no obvious mosaic or vein yellowing by 14 dpi (Table 1, Fig 3D). These symptoms became progressively milder by 21 dpi. In the host range experiment all *Abutilon* sp. plants (derived from seeds collected in the DO) agroinoculated with the infectious DNA-A and DNA-B components of AbGYMV were stunted and developed epinasty and striking golden/yellow mosaic of newly emerged leaves by 14 dpi (Fig 3E). Moreover, these symptoms were similar to those observed in *Abutilon* sp. plants in the DO (S1B Fig), thereby fulfilling Koch’s postulates for the golden/yellow mosaic disease of *Abutilon* sp. in the DO. In contrast, agroinoculated *Malachra* sp. plants developed no symptoms and only a small number of plants had DNA-A only infections (Table 1). AbGYMV induced mild upward leaf curling symptoms in *N*. *glutinosa* (Table 1), and very mild symptoms of leaf epinasty in common bean by 14 dpi (Fig 3F). Symptomless DNA-A and DNA-B infections were detected in agroinoculated *N*. *tabacum* (cvs. Havana and Turkish) and *D*. *stramonium* plants, whereas symptomless DNA-A only infections were detected in some tomato by 14 dpi (Table 1). AbGYMV did not infect Cayenne long pepper, pumpkin, *C*. *amaranticolor* and *A*. *indicum* plants.

In all these experiments, the presence of the inoculated DNA-A and DNA-B components was confirmed in newly emerged leaves of representative symptomatic and in all non-symptomatic plants by PCR tests with component-specific primers (S1 Table). Plants agroinoculated with the empty vector or bombarded with gold particles alone did not develop symptoms and were negative for the TbLCuCV/AbGYMV DNA-A and DNA-B components (Fig 3G–3J).

### Pseudorecombination experiments with cloned DNA components of TbLCuCV and AbGYMV

**Fig 4:**
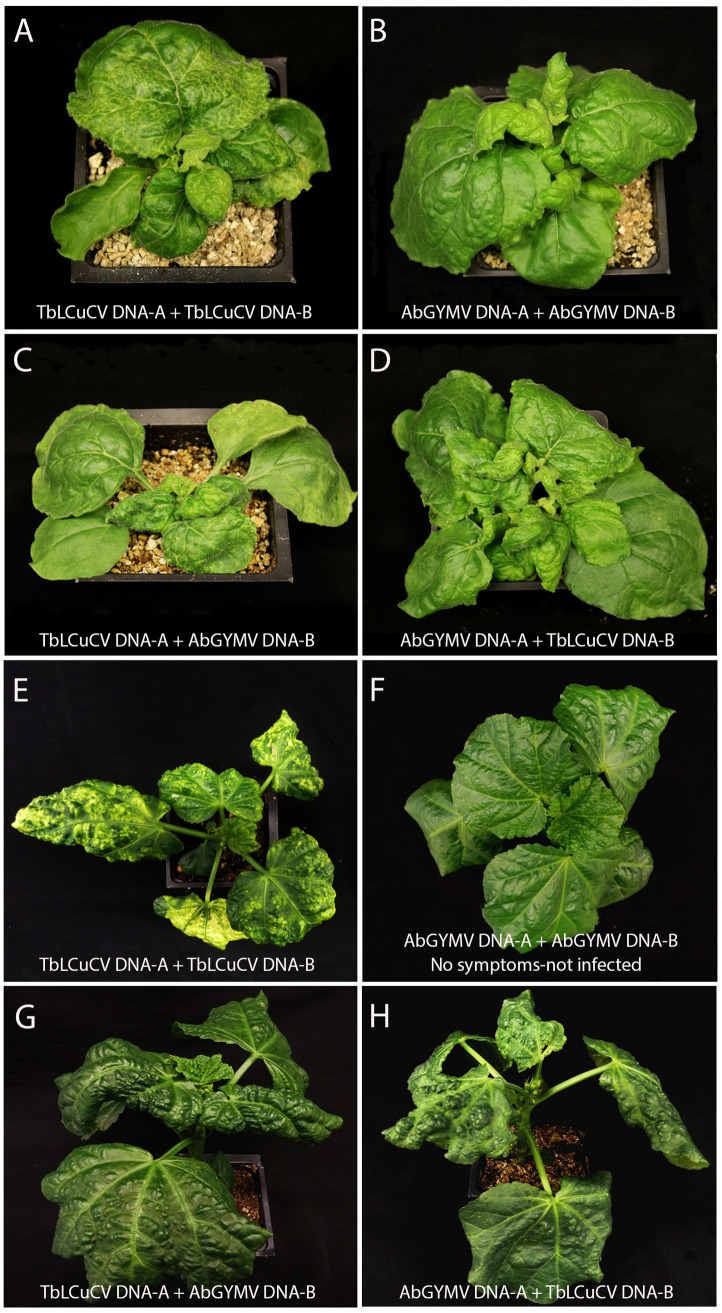
Disease symptoms induced by the pseudorecombinants (PRs) formed between the infectious cloned DNA-A and DNA-B components of an isolate of tobacco leaf curl Cuba virus from Haiti (TbLCuCV-[HT:14]) and the putative new bipartite begomovirus species Abutilon golden yellow mosaic virus from the Dominican Republic (AbGYMV-[DO:CG:16]) in: *Nicotiana benthamiana* (panels A-D) and *Malachra* sp. (panel E-H). Symptoms induced by TbLCuCV DNA-A and DNA-B are shown in panels A and E, whereas those induced by AbGYMV DNA-A and DNA-B are shown in panels B and F. Note that because AbGYMV did not infect *Malachra* sp. plants in these experiments, panel F shows an inoculated plant that did not become infected. Symptoms induced by TbLCuCV DNA-A (TA) and AbGYMV DNA-B (AbB) are shown in panels C and G, whereas those induced by AbGYMV DNA-A (AbA) and TbLCuCV DNA-B (TB) are shown in panels D and H. Plants were photographed 14 days after agroinoculation.

To further investigate the relationship between TbLCuCV and AbGYMV, pseudorecombination experiments were conducted in *N*. *benthamiana* and *Malachra* sp. plants (note that *Abutilon* sp. seeds from the DO were not available for these experiments) (Table 2). In *N*. *benthamiana*, pseudorecombinants (PRs) formed with the TbLCuCV DNA-A (TA) and AbGYMV DNA-B (AbB) or AbGYMV DNA-A (AbA) and TbLCuCV DNA-B (TB) were highly infectious (100%) and induced severe symptoms by 14 dpi. The TA + AbB PR induced epinasty, crumpling, deformation, mosaic and vein yellowing symptoms, which were more similar to those induced by the TbLCuCV parent (compared Fig 4A with 4C). In contrast, the AbA + TB PR induced mostly epinasty and crumpling symptoms, which were more similar to those induced by the AbGYMV parent (compared Fig 4B with 4D). Thus, the symptoms induced by these PRs were associated with the source of the DNA-A component. Furthermore, the symptoms induced by both PRs were more severe than those induced by the AbGYMV parent (compared Fig 4B with 4C and 4D). Taken together, these results suggest an important role for the DNA-A component in symptom development in this host.

**Table 2:** Infectivity and symptomatology of pseudorecombinants (PRs) formed with the infectious cloned DNA-A and DNA-B components of an isolate of tobacco leaf curl Cuba virus from Haiti (TbLCuCV-[HT:14]) and Abutilon golden yellow mosaic virus from the Dominican Republic (AbGYMV-[DO:CG:16]) following agroinoculation of *Nicotiana benthamiana* and *Malachra* sp. plants.

	Infectivity^b^				
Plant species/PRs^a^	TbLCuCV		AbGYMV		Symptoms^c^
	DNA-A	DNA-B	DNA-A	DNA-B	
***N*. *benthamiana***					
TA + AbB	9/9 (100)	0/9 (0)	0/9 (0)	9/9 (100)	Cr, E, Ld, M, S, Vy
AbA + TB	0/9 (0)	9/9 (100)	9/9 (100)	0/9 (0)	E, M, Ld, S
TA + TB	9/9 (100)	9/9 (100)	0/9 (0)	0/9 (0)	Cr, E, Ld, M, S, Vy
AbA + AbB	0/9 (0)	0/9 (0)	9/9 (100)	9/9 (100)	E, S
***Malachra* sp.**					
TA + AbB	7/9 (80)	0/9 (0)	0/9 (0)	7/9 (80)	Cr, E, Ld, S
AbA + TB	0/9 (0)	2/9 (22)	2/9 (22)	0/9 (0)	Cr, E, Ld, M, S
TA + TB	9/9 (100)	9/9 (100)	0/9 (0)	0/9 (0)	Cr, E, Ld, M, S
AbA + AbB	0/9 (0)	0/9 (0)	0/9 (0)	0/9 (0)	NS-ni

^a^ Virus abbreviation: TA, TbLCuCV DNA-A; TB, TbLCuCV DNA-B; AbA, AbGYMV DNA-A and AbB, AbGYMV DNA-B. PRs were formed by exchanging the infectious cloned DNA-A and DNA-B components of TbLCuCV and AbGYMV via agroinoculation of *N*. *benthamiana* and *Malachra* sp. plants. Controls were equivalent plants agroinoculated with the DNA-A and DNA-B components of TbLCuCV or AbGYMV (positive controls) and the empty vector alone (negative control).

^b^ Infectivity (number of infected plants/number inoculated) was determined at 14 days post inoculation based on symptom development and detection of viral DNA components by PCR tests with component-specific primer pairs; Percentages of infection is indicated in parentheses. Data represents a total of three independent experiments.

^d^ Symptom abbreviations: Cr, crumpling; E, epinasty; Ld, leaf deformation; M, mosaic/mottle; NS-ni, no symptoms-not infected; S, stunting; and Vy, vein yellowing.

In equivalent experiments conducted in *Malachra* sp., both PRs were infectious, but at lower rates than in *N*. *benthamiana*. Furthermore, the PRs differed markedly in infectivity, with the TA + AbB PR having an infection rate of 80%, whereas that of the AbA + TB was only 22%. The symptoms induced by these PRs were different compared with those induced by the parental viruses. Thus, both PRs induced more severe symptoms than those induced by the AbGYMV parent (compared Fig 4G and 4H with 4F). Furthermore, the TA + AbB PR induced epinasty, crumpling and deformation, but little yellow mosaic (Fig 4G); whereas the AbA + TB PR induced epinasty, crumpling, deformation as well as yellow mosaic by 14 dpi (Fig 4H). These results suggest an important role for the DNA-A component in infectivity and a role for the DNA-B component in symptom development in *Malachra* sp.

In PCR tests with component-specific primers, the inoculated DNA components were detected in newly emerged leaves of all symptomatic plants. Together, these results established that the components of these viruses are interchangeable, consistent with the conservation of critical CR sequences (e.g., iterons) and their close phylogenetic relationship (Figs 1 and 2). Moreover, infectivity and symptoms were host-dependent, involved both components and revealed evidence of differential adaptation of these viruses.

### Co-infection studies with TbLCuCV and AbGYMV

*Malachra* sp. plants were co-agroinoculated with the infectious cloned DNA-A and DNA-B components of TbLCuCV and AbGYMV (four component inoculation) to determine if these viruses can co-infect this species. Here, 80% of *Malachra* sp. plants co-agroinoculated with these components were stunted and newly emerged leaves showed epinasty, crumpling, deformation and golden/yellow mosaic by 14 dpi (Table 3). These symptoms were indistinguishable from those induced by the TbLCuCV DNA-A and DNA-B components in *Malachra* sp. (Fig 3B). PCR tests with component-specific primers revealed that all of the symptomatic plants were infected with the TbLCuCV DNA-A and DNA-B components, whereas the AbGYMV DNA-A and DNA-B components were detected in 22% and 33% of these plants, respectively. These results indicate that TbLCuCV and AbGYMV can co-infect *Malachra* sp. and that TbLCuCV may enhance infectivity of AbGYMV in *Malachra* sp., but there was no evidence of synergism in terms of disease symptoms.

**Table 3:** Infectivity and symptomatology in *Malachra* sp. plants co-agroinoculated with the infectious cloned DNA-A and DNA-B components of an isolate of tobacco leaf curl Cuba virus from Haiti (TbLCuCV-[HT:14]) and Abutilon golden yellow mosaic virus from the Dominican Republic (AbGYMV-[DO:CG:16]).

	Infectivity^b^				
Plant species/Combination^a^	TbLCuCV		AbGYMV		Symptoms^c^
	DNA-A	DNA-B	DNA-A	DNA-B	
TA + TB + AbA + AbB	7/9 (80)	7/9 (80)	2/9 (22)	3/9 (33)	Cr, E, Ld, M, S
TA + TB	5/5 (100)	5/5 (100)	0/9 (0)	0/9 (0)	Cr, E, Ld, M, S
AbA + AbB	0/9 (0)	0/9 (0)	0/9 (0)	0/9 (0)	Ni-ni
Empty vector	0/9 (0)	0/9 (0)	0/9 (0)	0/9 (0)	Ni-ni

^a^ Abbreviation: TA, TbLCuCV DNA-A; TB, TbLCuCV DNA-B; AbA, AbGYMV DNA-A; AbB, AbGYMV DNA-B. Experiments were performed by agroinoculation of the infectious cloned DNA-A and DNA-B components of TbLCuCV and AbGYMV in *Malachra* sp. plants. Controls were equivalent plants agroinoculated with the DNA-A and DNA-B components of TbLCuCV or AbGYMV (positive controls) and the empty vector alone (negative control).

^b^ Infectivity (number of infected plants/number inoculated) was determined at 14 days post inoculation based on symptom development and detection of viral DNA components by PCR tests with component-specific primers; Percentages of infection is indicated in parentheses. Data represents a total of three independent experiments.

^c^ Symptom abbreviations: Cr, crumpling; E, epinasty; Ld, leaf deformation; M, mosaic/mottle; NS-ni, no symptoms-not infected and S, stunting.

## Discussion

In the present study, we determined the etiology of golden/yellow mosaic diseases of two malvaceous weed species in Hispaniola, as part of a long-term project to characterize begomoviruses infecting weeds and determine their potential to cause diseases of crop plants in the DO. We used DNA barcoding to identify the larger malva-like weed as *Malachra* sp. and the smaller bushy plant as *Abutilon* sp. *Malachra* sp. is an invasive weed found in association with irrigation ditches and disturbed areas around agricultural fields in Hispaniola and other countries of the Caribbean Basin, whereas *Abutilon* sp. also occurs in this environment, but was less common based in our surveys.

Infection of *Malachra* sp. by TbLCuCV in HT and the DO is the first report of this virus in these countries, and extends on a previous report of TbLCuCV infecting *M*. *alceifolia* in JM [36]. Together with reports of TbLCuCV infecting tobacco and common bean in CU Morán et al. 2006; Leyva et al. 2016) [71,72], it appears that this virus is widely distributed in the Caribbean Basin, where it infects crops and weeds. Infectivity and host range experiments with infectious clones confirmed that TbLCuCV induces golden/yellow mosaic symptoms in *Malachra* sp. and stunting and epinasty, crumpling and mosaic/mottling in tobacco and common bean, thereby fulfilling Koch’s postulates for these diseases. This raises the question of what is the prevalent host of this virus in the Caribbean Basin, and several lines of evidence suggest it may be *Malachra* sp. First, TbLCuCV has now been detected in *Malachra* spp. in multiple countries in the Caribbean Basin, whereas disease outbreaks in crops (tobacco and common bean) have only been reported from CU and seem to be sporadic [71,72]. Second, TbLCuCV is most closely related to other weed-infecting bipartite begomoviruses from the Caribbean Basin (Fig 2), consistent with evolution of these viruses from a common ancestor and adapted to non-cultivated (weed) species. Third, numerous weed-infecting begomoviruses have been reported to also infect and cause disease symptoms in crop plants under laboratory conditions, but such diseases are rarely observed in the field (Domínguez et al. 2009; Morán et al. 2006; Melgarejo et al. 2014; Barbosa et al. 2011; Blawid et al. 2013; Pramesh et al. 2013; Tahir et al. 2015) [20,34,71,73–76]. In this scenario, *Malachra* sp. is the prevalent host of TbLCuCV and, under certain conditions, e.g., high whitefly populations, the virus can spill over into crops and causes disease outbreaks. Thus, although TbLCuCV can infect crop species, it is not a *bona fide* crop-infecting begomovirus such as BGYMV, which is highly adapted to common bean and is rarely detected in weeds [77]. Finally, we hypothesize that the golden/yellow mosaic disease of *Malachra* sp. caused by TbLCuCV also occurs in CU and may be the source of inoculum for outbreak in crops.

A different disease etiology and biology was determined for the golden/yellow mosaic disease of *Abutilon* sp. in the DO. Here, we identified a new bipartite begomovirus, AbGYMV, in samples of plants with these symptoms collected in two location in the DO. We further showed that the infectious clones of this virus DNA components of this virus induced striking golden/yellow mosaic symptoms in plants of *Abutilon* sp. from the DO, thereby fulfilling Koch’s postulates for this disease. Furthermore, host range experiments showed that AbGYMV is highly adapted to this species of *Abutilon* sp. from the DO, as it induced mild or no symptoms in other species tested, e.g., *N*. *benthamiana*, common bean, tobacco, *Malachra* sp., and *A*. *indicum* from India. These results may indicate a long period of virus-host co-evolution, with the interaction having reached an equilibrium. Evidence for this comes from the nature of the disease symptoms, which were mostly striking golden/yellow mosaic with little or no distortion, crumpling or curling of leaves. A similar situation has been described for the striking golden/yellow mosaic symptoms induced by AbMV in *A*. *hybridum* [42,78]. AbMV is the NW bipartite begomovirus for which the AbMV lineage was named and it originated in the Caribbean Basin (West Indies). Furthermore, years of graft-transmission (>150 years) has resulted in the selection of abutilon plants showing only the striking golden/yellow mosaic disease (i.e., virus-host equilibrium). Indeed, these symptoms are so aesthetically pleasing that these plants are sold commercially as a variegated variety of flowering maple [42,78].

In the phylogenetic analyses, TbLCuCV and AbGYMV were placed together as sister groups in a strongly supported clade, consistent with evolution from a common ancestor followed by diversification driven through host adaptation. The long branch separating AbGYMV from the clade ancestor further suggests a relatively long period of evolution, which often indicates recombination [9,79]. Indeed, a recombination event was detected in the well-known hot-spot region of the AbGYMV DNA-A component [13,69,70,79]. Furthermore, the high degree of sequence divergence across the genomes of TbLCuCV and AbGYMV, including in the CR and HVR, are consistent with evolution via mutation, another major mechanism of begomovirus diversification (Lima et al. 2017; Duffy et al. 2008) [9,80]. The common ancestor of TbLCuCV and AbGYMV was probably a bipartite begomovirus infecting non-cultivated plants on Hispaniola. Subsequent local evolution and host adaptation, presumably driven by indigenous whiteflies, led to the emergence of viruses adapted to non-cultivated weed species, such as *Malachra* sp. and *Abutilon* sp., respectively. This is supported by two lines of evidence. First, TbLCuCV and AbGYMV are most closely related to each other and to the C1 clade of the AbMV lineage, which contains bipartite weed-infecting begomoviruses from the Caribbean Basin (e.g., JMV and WGMV) (Figs 2 and S2). Second, the results of the infectivity studies showed that TbLCuCV and AbGYMV are well-adapted to *Malachra* sp. and *Abutilon* sp., respectively. It is also worth noting that it is not clear weather *Malachra* sp. and *Abutilon* sp. are native or introduced species on Hispaniola.

The capacity of these bipartite begomoviruses to form infectious PRs can provide insight into relatedness and gene function [8,12,13,16,34,81–86]. The highly infectious nature of the PRs formed between the DNA-A and DNA-B components of TbLCuCV and AbGYMV likely reflects the importance of the conserved CR iterons, because there was substantial divergence in the CR sequence outside of these elements (Fig 1). Furthermore, the Rep IRDs of these viruses are different, with the TbLCuCV IRD lacking the highly conserved F residue and not predicted to recognize the “GGGGG” core iteron [68]. In the case of the IRD, the substitution of the aromatic non-polar F residue with a polar S residue suggests a non-essential role for the aromatic side chain and some shared biochemical properties between these aa that allow IRD function. Thus, this is another example of unexpected genetic flexibility in the IRD-iteron interactions revealed by the infectivity of PRs formed between viruses from different lineages (Garrido-Ramirez et al. 2000) [16]. Moreover, an efficient interaction between components of these viruses was revealed by the highly infectious and virulent TbLCuCV/AbGYMV PRs in *N*. *benthamiana* and *Malachra* sp., despite the CR/IRD differences. In fact, both PRs were more virulent than the AbGYMV parent, which is atypical for PRs. This may be explained in terms of the host adaptation and co-evolution of AbGYMV with the local species of *Abutilon* sp. In this regard, AbGYMV may have become so well-adapted to this *Abutilon* sp. that it is not well suited to infect and induce symptoms in *N*. *benthamiana* and *Malachra sp*. However, this equilibrium in the virus-host interaction can be disrupted when PRs are formed with the TbLCuCV DNA components. Thus, the higher virulence of both PRs may be due to the uncoupling of aspects of the host adaptation of the AbGYMV DNA-A and DNA-B components when combined with the components of TbLCuCV.

The disease phenotypes of the PRs also revealed that infectivity and pathogenicity determinants mapped to both DNA components and in a host-dependent manner. In *N*. *benthamiana*, the symptom phenotypes were associated with the source of the DNA-A component, whereas in *Malachra* sp. the DNA-A component played an important role in infectivity and the DNA-B component was associated with the symptom phenotype (Table 2). These results are in agreement with previous studies showing that host specificity and symptom development are complex phenomena that involves interaction among multiple virus- and host-encoded factors as well as non-translated regions [13,87–91]. Thus, there are numerous examples of DNA-A sequences/gene products being symptoms determinants [13,87,88,91], possibly involving virus-host interactions associated with replication, gene expression or suppression of host defenses. The very low rate of infectivity of AbA + TB in *Malachra* sp. may be related to early establishment of infection in this host, e.g., capacity for movement or defense suppression, rather than a deficiency of replication, because some AbA + TB plants developed severe symptoms and had wild-type DNA levels based on semi-quantitative PCR test results. This low rate of infectivity also cannot be explained by incompatibility of the DNA-B-encoded proteins, as the PRs induced symptoms as severe or more so than the parental viruses. Thus, the low infectivity in *Malachra* sp. may reflect differences in interactions with host factors, such as those involved in gene expression or silencing mediated by the AbA-encoded proteins, including expression of the TB-encoded NSP and MP. In *Malachra* sp., the role of the DNA-B component in symptom development is in agreement with previous studies that showed both DNA-B encoded proteins are symptom determinants [1,8,12,13,16,81,85,86,88]. Indeed, these aspects of the TbLCuCV/AbGYMV interaction may make this system useful for further mapping the host-specific role of the DNA-B encoded protein in symptom development.

In conclusion, we showed that the etiology of the golden/yellow mosaic diseases of two malvaceous species in Hispaniola are caused by TbLCuCV and AbGYMV, respectively. TbLCuCV also infects common bean or tobacco, consistent with causing occasional disease outbreaks, whereas AbGYMV induces few or no symptoms in crop plants. TbLCuCV and AbGYMV are closely related viruses in the AbMV lineage of NW begomoviruses, and can form infectious PRs and coinfect *Malachra* sp. plants, which allows for further virus evolution. Finally, our results indicated that TbLCuCV and AbGYMV do not appear to pose a threat to crop production in the DO, although TbLCuCV-infected *Malachra* sp. could serve as sources of inoculum for sporadic spillover outbreaks in crops, such as in tobacco grown in the Cibao Valley in the northern DO.

## Supporting information

S1 Fig (A) *Malachra* sp. plant with leaf crumpling and yellow vein and mosaic/mottle symptoms associated with infection by the New World bipartite begomovirus, tobacco leaf curl Cuba virus (TbLCuCV) in the Dominican Republic (DO) in 2016. (B) *Abutilon* sp. plant with yellow mosaic/mottle symptoms associated with infection by Abutilon golden yellow mosaic virus (AbGYMV), a putative new bipartite begomovirus species, in Cerro Gordo, DO in 2020.
(TIF)

S2 Fig Map of the island of Hispaniola showing the geographic locations where malvaceous weeds with golden/yellow mosaic symptoms were collected from Port-Au-Prince, Haiti in 2014; from three locations in the Dominican Republic (DO) in 2016: Juan Gomez, Manzanillo and Cerro Gordo; and from two locations in the DO in 2020: Cerro Gordo and Monte Cristi. (TIF)

S3 Fig Bayesian phylogenetic consensus tree constructed with complete nucleotide sequences of the DNA-B components and showing the relationship of three isolates of tobacco leaf curl Cuba virus (TbLCuCV) from Hispaniola and the putative new bipartite begomovirus species Abutilon golden yellow mosaic virus (AbGYMV) from the Dominican Republic (shown in bold) with: (i) the most closely related begomoviruses identified based on a BLASTn search and (ii) selected begomoviruses representing the Abutilon mosaic virus (AbMV), Brazil, squash leaf curl virus (SLCuV), bean golden yellow mosaic virus (BGYMV) and Boerhavia golden mosaic virus (BoGMV) lineages of New World (NW) bipartite begomoviruses (lineages are indicated with brackets). The two major clades of the AbMV lineage, C1 and C2, as well as the paraphyletic group C3 are shown with inner brackets. Sequences were obtained from GenBank, and virus abbreviations are as described in Brown et al. 2015. Branch strengths were evaluated by Bayesian posterior probabilities. The phylogenetic consensus tree was rooted with the complete sequence of the DNA-B component of the Old World bipartite begomovirus African cassava mosaic virus (ACMV). The length of horizontal branches indicates the rate of substitution per nucleotide.
(TIF)

S4 Fig Schematic representation of the recombination event identified in the DNA-A component of Abutilon golden yellow mosaic virus from the Dominican Republic (AbGYMV-[DO:CG:16]) with the recombination detection program version 4.0 (RDP4). This strongly supported recombination event was 625 nucleotides (nts), spans nts 1994 to 2618 and includes the 5’ end of the AC1 open reading frame (ORF), the entire overlapping AC4 ORF and the left side of the common region. This event is in the well-known recombination hot-spot in the begomovirus genomic DNA/DNA-A component. Furthermore, RDP4 indicated that the recombinant region came from an unidentified minor parent, and that the major parent was tomato yellow leaf distortion virus (ToYLDV) (GenBank accession number FJ174698). The CR is represented by a grey curved box and viral ORFs are represented by arrows.
(TIF)

S1 Table Sequences of the oligonucleotide primers used in this study. (PDF)

S2 Table Nucleotide (nt) identities for total (Tot) and common region (CR) and hypervariable region (HVR) sequences and nt and amino acid (aa) identities and similarities (in parenthesis) of individual open reading frames (ORFs) for the DNA-A and DNA-B components of an isolate of tobacco leaf curl Cuba virus from Haiti (TbLCuCV-[HT:14]) with TbLCuCV isolates from the Dominican Republic and Cuba and the most closely related begomoviruses. (PDF)

S3 Table Nucleotide (nt) identities for total (Tot) and common region (CR) and hypervariable region (HVR) sequences and nt and amino acid (aa) identities and similarities (in parenthesis) of individual open reading frames (ORFs) for the DNA-A and DNA-B components of Abutilon golden yellow mosaic virus from the Dominican Republic (AbGYMV-[DO:CG:16]) and the most closely related begomoviruses. (PDF)
