# Supplementary figures and images for: Weed-infecting viruses in a tropical agroecosystem present different threats to crops and evolutionary histories
Source: PLoS One. 2021 Apr 28;16(4):e0250066. doi: 10.1371/journal.pone.0250066 (PMC8081230; doi:10.1371/journal.pone.0250066)

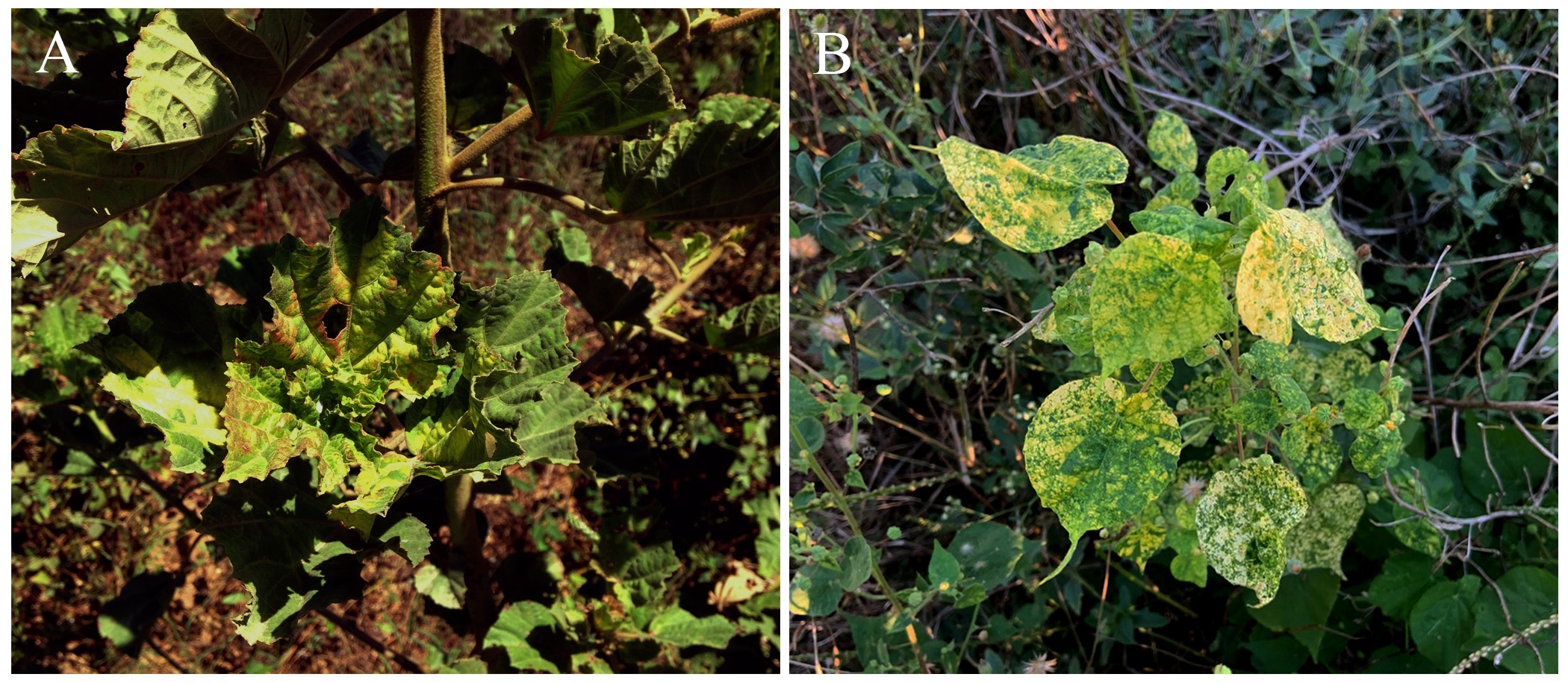

Supplement: S1 Fig — (A) Malachra sp. plant with leaf crumpling and yellow vein and mosaic/mottle symptoms associated with infection by the New World bipartite begomovirus, tobacco leaf curl Cuba virus (TbLCuCV) in the Dominican Republic (DO) in 2016. (B) Abutilon sp. plant with yellow mosaic/mottle symptoms associated with infection by Abutilon golden yellow mosaic virus (AbGYMV), a putative new bipartite begomovirus species, in Cerro Gordo, DO in 2020. (TIF) [file pone.0250066.s001.tif]

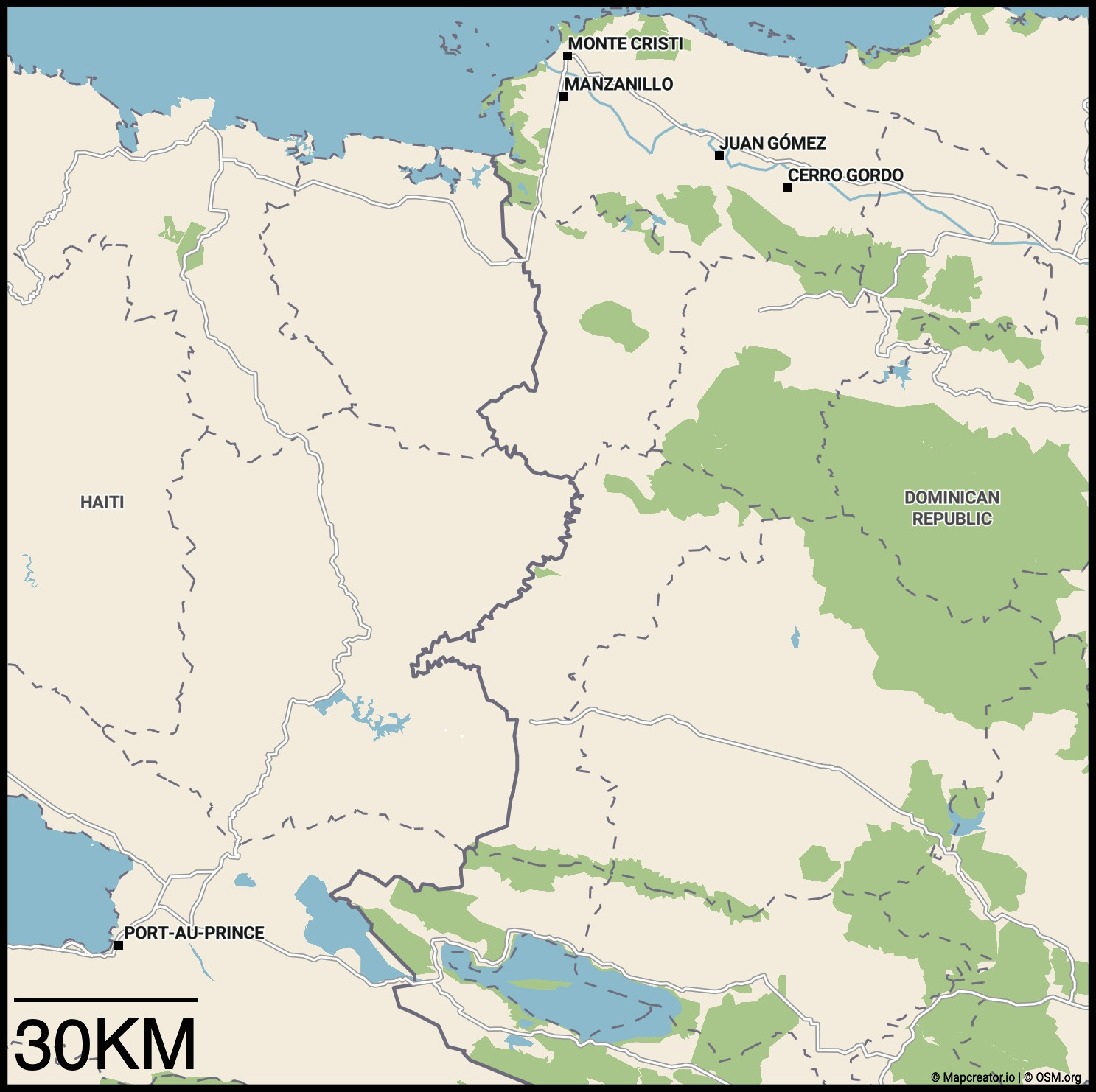

Supplement: S2 Fig — (TIF) [file pone.0250066.s002.tif]

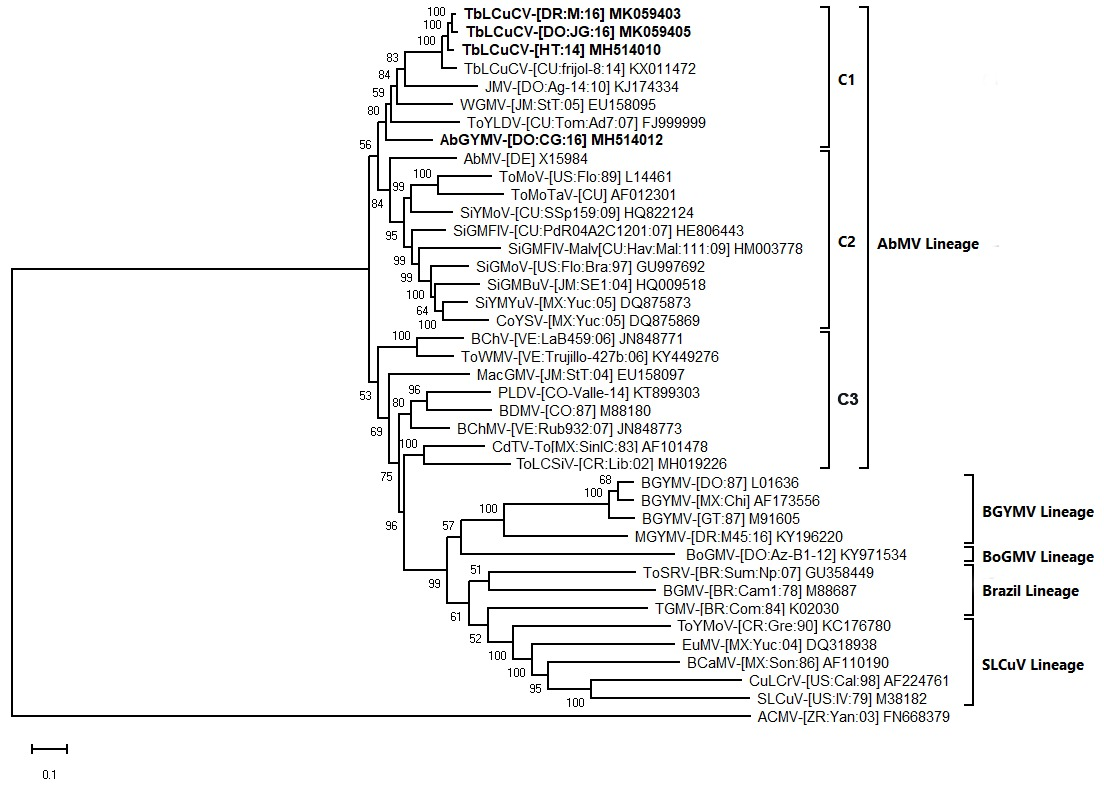

Supplement: S3 Fig — The two major clades of the AbMV lineage, C1 and C2, as well as the paraphyletic group C3 are shown with inner brackets. Sequences were obtained from GenBank, and virus abbreviations are as described in Brown et al. 2015. Branch strengths were evaluated by Bayesian posterior probabilities. The phylogenetic consensus tree was rooted with the complete sequence of the DNA-B component of the Old World bipartite begomovirus African cassava mosaic virus (ACMV). The length of horizontal branches indicates the rate of substitution per nucleotide. (TIF) [file pone.0250066.s003.tif]

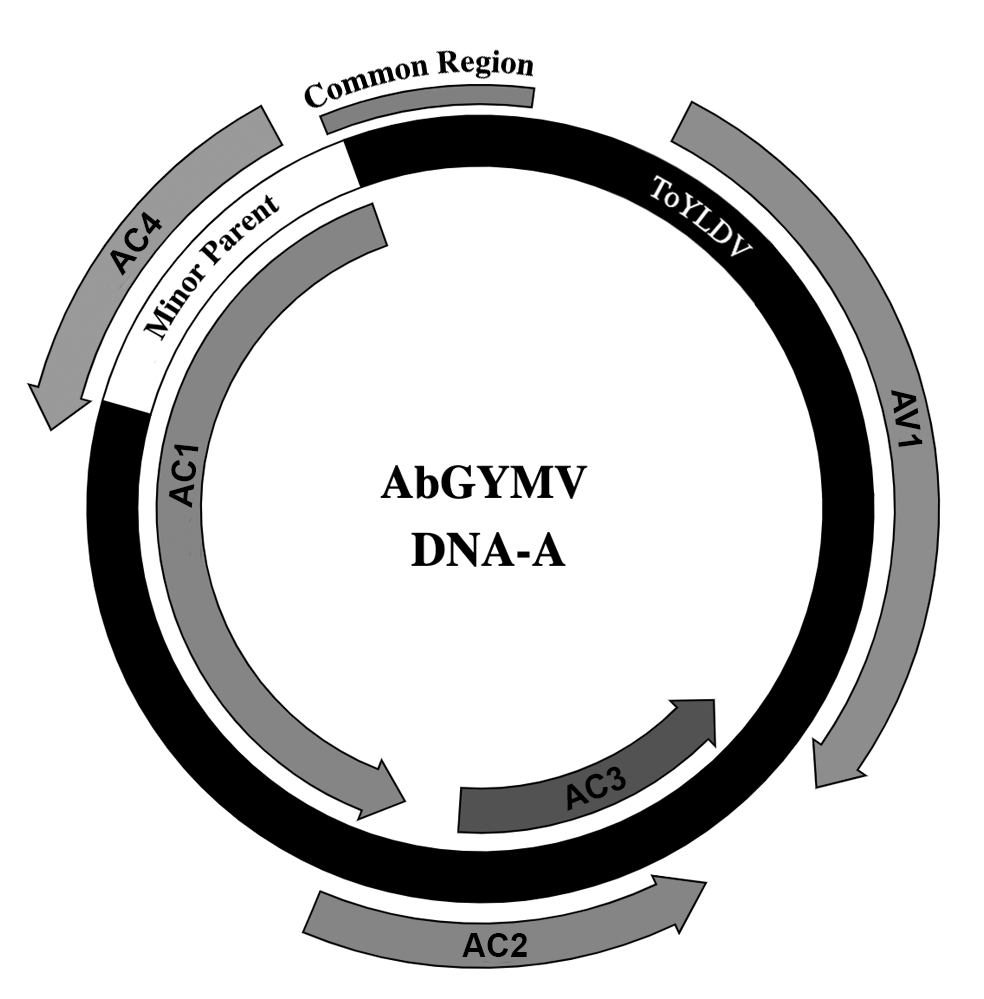

Supplement: S4 Fig — This strongly supported recombination event was 625 nucleotides (nts), spans nts 1994 to 2618 and includes the 5’ end of the AC1 open reading frame (ORF), the entire overlapping AC4 ORF and the left side of the common region. This event is in the well-known recombination hot-spot in the begomovirus genomic DNA/DNA-A component. Furthermore, RDP4 indicated that the recombinant region came from an unidentified minor parent, and that the major parent was tomato yellow leaf distortion virus (ToYLDV) (GenBank accession number FJ174698). The CR is represented by a grey curved box and viral ORFs are represented by arrows. (TIF) [file pone.0250066.s004.tif]
