# Supplementary material for: Weed-infecting viruses in a tropical agroecosystem present different threats to crops and evolutionary histories
Source: PLoS One. 2021 Apr 28;16(4):e0250066. doi: 10.1371/journal.pone.0250066 (PMC8081230; doi:10.1371/journal.pone.0250066)
Supplement: S1 Table — (PDF) [file pone.0250066.s005.pdf]

Table S1. Sequences of the oligonucleotide primers used in this study

Primer[a]	Nucleotide sequence
PACUv2353	5'-CGGAGTATTTACTTTCTGTAATTGGG-3´
PACUv1889	5'-GGAACGTTAGTGAAAGAGGAGAGTTG-3´
PACUc174	5'-GTCCATACAGGCCCAAACAGCTTAAA-3´
PBCUv2171	5'-ATTCGGGAGAAGAAGAGAGGAATAG-3´
PBCUv1779	5'-GATCTATGTTGCATCTGATCGG-3´
PBCUc159	5'-CAAATTGTCAGGCAGAAATCGG-3´
PADRv1980	5'-AATCCTATCGAGATTTGCCCTTAG-3´
PADRc205	5'-TTAAAGAAAAGGGACCAATCAGCTTTTC-3´
PADRv1918	5'-GGCACGTTAGTAAAAGAGGAGAGTTG-3´
PADRc2402	5'-TTACTTAAACTCCAAACCCCATCCAAG-3´
PBDRv2241	5'-GCGTTGGATAAGATGGAAGAAGG-3´
PBDRc167	5'-GTAAAACAATTCGCGCGACAGAAG-3´

[a] Primer nomenclature is as follows: P, primer; A, DNA-A component; B, DNA-B component; CU, TbLCCuV; DR, AbGYMV; v, viral sense primer; c, complementary sense primer; and number of the annealing position for the 5' end of the primer.
