# Supplementary material for: Weed-infecting viruses in a tropical agroecosystem present different threats to crops and evolutionary histories
Source: PLoS One. 2021 Apr 28;16(4):e0250066. doi: 10.1371/journal.pone.0250066 (PMC8081230; doi:10.1371/journal.pone.0250066)
Supplement: S2 Table — (PDF) [file pone.0250066.s006.pdf]

Table S2. Nucleotide (nt) identities for total (Tot) and common region (CR) and hypervariable region (HVR) sequences and nt and amino acid (aa) identities and similarities (in parenthesis) of individual open reading frames (ORFs) for the DNA-A and DNA-B components of an isolate of tobacco leaf curl Cuba virus from Haiti (TbLCuCV-[HT:14]) with TbLCuCV isolates from the Dominican Republic and Cuba and the most closely related begomoviruses[a]

		DNA-A														DNA-B[d]					
				ORFs												ORFs			
				AV1		AC1		AC2		AC3		AC4				BV1		BC1	
Species[b]	Loc[c]	Tot	CR	nt	aa	nt	aa	nt	aa	nt	aa	nt	aa	Tot	HVR	nt	aa	nt	aa
TbLCuCV-[DO:JG:16]	DO	97	98	97	99 (100)	98	97 (98)	98	98 (99)	96	97 (96)	99	99 (98)	97	92	98	98 (99)	97	99 (99)
TbLCuCV-[DO:M:16]	DO	97	92	97	99 (100)	98	98 (99)	99	98 (99)	97	98 (98)	99	99 (100)	97	93	98	99 (99)	98	99 (99)
TbLCuCV-[CU:VC-CU2015:14]	CU	96	94	96	98 (99)	96	97 (98)	99	98 (99)	98	98 (98)	98	95 (94)	NA	NA	NA	NA	NA	NA
TbLCuCV-[CU:frijol-8:14]	CU	96	92	96	99 (100)	97	96 (98)	99	98 (99)	98	98 (99)	98	96 (95)	94	91	96	96 (98)	95	98 (99)
TbLCuCV-[CU:Tag:05][e]	CU	96	93	96	98 (99)	96	97 (97)	98	98 (99)	98	98 (98)	*	*	NA	NA	NA	NA	NA	NA
TbMoLCV -[CU:SSp:03]	CU	87	93	87	92 (95)	87	83 (86)	91	76 (80)	91	89 (92)	92	86 (86)	NA	NA	NA	NA	NA	NA
SiYMoV-[CU:SSp159:09]	CU	87	85	88	96 (98)	88	87 (93)	91	88 (90)	91	91 (93)	91	82 (81)	78	59	77	75 (87)	88	95 (97)
WGMV-[JM:Alb:08]	JM	87	80	89	90 (94)	86	86 (90)	90	91 (94)	93	92 (93)	88	82 (81)	81	71	81	83 (88)	90	95 (97)
JMV-[JM:ST1:04]	JM	87	89	89	92 (96)	86	84 (91)	90	86 (90)	91	89 (92)	91	79 (80)	79	68	81	79 (86)	86	94 (96)
SiGYVV-[US:Sa19:12]	US	86	81	86	91 (95)	87	87 (91)	91	90 (92)	90	87 (89)	90	80 (81)	76	59	75	73 (83)	84	90 (95)
AbGYMV-[DO:CG:16]	DO	86	85	88	94 (98)	84	84 (90)	93	89 (92)	91	90 (94)	86	66 (74)	82	58	85	86 (91)	89	96 (98)
ToYLDV-[CU:5E17:07]	CU	86	87	84	88 (95)	85	83 (89)	93	89 (94)	93	90 (92)	88	76 (78)	80	59	82	80 (88)	87	92 (96)
OYMV-[MX:Maz3:04]	MX	86	76	88	94 (98)	86	88 (92)	90	84 (87)	90	90 (94)	87	69 (77)	74	60	72	70 (82)	84	92 (96)
MaYMJV-[JM:9bA43:03]	JM	85	82	85	92 (96)	86	85 (90)	90	84 (86)	90	90 (90)	87	74 (76)	74	58	77	75 (83)	82	90 (96)
RhRGMV-[CU:Cam:171:09]	CU	85	87	87	95 (97)	83	82 (89)	91	88 (90)	90	90 (93)	88	79 (81)	70	57	73	68 (82)	76	86 (89)

[a] Based on a BLASTn analysis, the most closely related begomoviruses were various New World bipartite begomoviruses.

[b] GenBank accession numbers are as follows: TbLCuCV-[HT:14]: MH514009 and MH514010; TbLCuCV-[DO:JG:16]: MK059404 and MK059405; TbLCuCV-[DO:M:16]: MK059402 and MK059403; TbLCuCV-[CU:VC-CU2015:14]: KU562963; TbLCuCV-[CU:frijol-8:14]: KX011471 and KX011472; TbLCuCV-[CU:Tag:05]: AM050143; TbMoLCV -[CU:SSp:03]: NC038893; SiYMoV-[CU:SSp159:09]: HQ822123 and HQ822124; WGMV-[JM:Alb:08]: GQ355488 and GQ355487; JMV-[JM:ST1:04]: KF723258 and KF723261; SiGYVV-[US: Sa19:12]: KT879816 and KT879818; AbGYMV-[DO:CG:16]: MH514011 and MH514012; ToYLDV-[CU:5E17:07]: FJ174698 and NC017913; OYMV-[MX:Maz3:04]: DQ022611 and GU972604; MaYMJV-[JM:9bA43:03]: FJ600482 and FJ600484 and RhRGMV-[CU:Cam:171:09]: NC038805 and NC038804.

[c] Geographic location: DO = Dominican Republic, CU = Cuba, JM = Jamaica, US = United States and MX= Mexico.

[d] NA = not available.

[e] *ORF AC4 is truncated.
